# Correcting Errors in Image Encryption Based on DNA Coding

**DOI:** 10.3390/molecules23081878

**Published:** 2018-07-27

**Authors:** Bin Wang, Yingjie Xie, Shihua Zhou, Xuedong Zheng, Changjun Zhou

**Affiliations:** 1 Key Laboratory of Advanced Design and Intelligent Computing, Dalian University, Ministry of Education, Dalian 116622, China; shihuajo@gmail.com (S.Z.); xuedongzheng@163.com (X.Z.); 2 Applied Technology College, Dalian Ocean University, Dalian 116300, China; yingjieying@163.com; 3 College of Mathematics, Physics and Information Engineering, Zhejiang Normal University, Jinhua 321004, China

**Keywords:** image encryption, chaotic map, DNA coding, Hamming distance

## Abstract

As a primary method, image encryption is widely used to protect the security of image information. In recent years, image encryption pays attention to the combination with DNA computing. In this work, we propose a novel method to correct errors in image encryption, which results from the uncertainty of DNA computing. DNA coding is the key step for DNA computing that could decrease the similarity of DNA sequences in DNA computing as well as correct errors from the process of image encryption and decryption. The experimental results show our method could be used to correct errors in image encryption based on DNA coding.

## 1. Introduction

With wide usage of multimedia technologies and excessive spread of internet, the awareness of protecting information, especially image information, is heightened day by day. As we known, encrypting technology can usually be used to protect the security of image information. In image encryption, chaotic maps are usually employed to encrypt image, because they have the features of ergodicity, sensitivity to initial conditions, control parameters and so on [1,2,3,4,5,6,7]. Chen et al. proposed a novel 3D cat maps to design a real-time secure symmetric encryption scheme [1]. Lian et al. first analyzed the parameter sensitivity of standard map and proposed an improved standard map to encrypt image [3]. Wong et al. proposed a fast algorithm of image encryption, where the overall encryption time was reduced as fewer rounds were required [2]. Zhang et al. proposed a new image encryption algorithm based on the spatiotemporal chaos of the mixed linear-nonlinear coupled map lattices [7]. Wang et al. combined genetic recombination with hyper-chaotic system to design a novel image encryption and experiment results proved that the proposed algorithm was effective for image encryption [8]. Zhang et al. analyzed different kinds of permutation algorithms and proposed a new cryptosystem to address these drawbacks [5]. In the recent past, although these methods have made some progress, they lack the capability of parallel computing.

Inspired by the biological character of DNA sequences, such as parallel computing, low-energy and so on, DNA computing and DNA coding are widely used to encrypt image [6,9,10,11,12,13,14,15,16,17]. Zhang et al. combined DNA sequence addition operation with chaotic map to design a novel image encryption scheme [9]. The experimental results shown that the proposed scheme could achieve good encryption and resist some kind attacks. In Ref. [10], the authors transformed DNA sequences into its base pair for random times to confuse the pixels, generate the new keys according to the plain image and the common keys. Wei et al. further utilized DNA sequence addition operation and Chen’s hyper-chaotic map to encrypt a color image [11]. Due to some disadvantages in One-Time-Pad (OTP) algorithm, the author used logistic chaotic map as an input of OTP algorithm and proposed an interesting encryption algorithm based on a chaotic selection between original message DNA strands and OTP DNA strands [12]. In Ref. [13], the authors used genetic algorithm to determine the best masks, which result from DNA and logistic map functions. Ozkaynak et al. broke a previous cryptosystem and proposed an improved image encryption algorithm [14]. Rehman et al. utilized whole set of DNA complementary rules dynamically and employed DNA addition operation to encrypt image [15]. Song and Qiao proposed a novel image encryption scheme based on DNA encoding and spatiotemporal chaos, which was of high key sensitivity and large key space [16]. In Ref. [17], DNA coding combined with an improved 1D chaotic systems to design image encryption. Kulsoom et al. employed an entire set of DNA complementary rules along with 1D chaotic maps to design an image encryption algorithm [6]. Wang et al. proposed a new chaotic image encryption scheme based on Josephus traversing and mixed chaotic map [18]. Parvaz and Zarebnia defined a combination chaotic system and studied its properties [19].

DNA computing was addressed to solve the seven-point Hamiltonian path problem by Adleman in 1994 [20]. Along with the development of research, there are a large number of applications about DNA computing, such as DNA logic gates [21], neural network [22], cryptography [4], data storage [23], image watermarking [24] and so on. Hybridization reaction is the key operation for DNA sequences and influences the reliability of DNA computing. However, the false hybridization is unavoidable because of the limit of biological technology, result from false positive and false negative. The lack of similarity between DNA sequences could result in false positive and generating hybridization reaction between two unmatched DNA sequences. The mistake in the biochemical operation result in false negative in which two matched DNA sequences did not hybridize each other [25]. Chai et al. encoded plain image by DNA matrix and permuted the image with a new wave-based permutation scheme [26]. In Ref. [27], DNA sequence operation combining with one-way coupled-map lattices was to structure a robust and lossless color image encryption algorithm and the three gray-level components of plain-image were converted into three DNA matrices and performed XOR operation twice. Designing DNA coding could obtain high quality DNA sequences which satisfy some constraints, such as Hamming distances, GC content and so on, to decrease the similarity between DNA sequences [28,29]. Inspired by Hybridization reaction is the kernel for DNA computing and influences the reliability of DNA computing. However, the false hybridization is unavoidable because of the limit of biological technology, result from false positive and false negative. The lack of similarity between DNA sequences could result in false positive and generating hybridization reaction between two unmatched DNA sequences. The mistake in the biochemical operation result in false negative in which two matched DNA sequences did not hybridize each other [25]. Designing DNA coding could obtain high quality DNA sequences which satisfy some constraints, such as Hamming distance, GC content and so on, to decrease the similarity between DNA sequences [28,29]. Inspired by communication theory, Hamming code can be used to correct errors. For example, *d* is the Hamming distance between two strings and then the bits of correcting errors are equal to $\left\lfloor \frac{d-1}{2}\right\rfloor$. So, in this paper, we introduce Hamming distance to decrease the similarity between DNA sequences as well as correct errors from hybridization reaction. Furthermore, to improve the accuracy of DNA computing, the constraints of DNA coding are used to decrease the generation of false positive. Finally, the experimental results show that the number of pixels change rate (NPCR) has achieved 99.57% and the unified average changing intensity (UACI) has achieved 32.38%. The proposed method could effectively correct the encrypted image contained 1000 errors and improve the accuracy of hybridization reaction.

## 2. Methods

### 2.1. DNA Coding

Hamming distance is widely used to design DNA coding. It is the number of positions at which the corresponding symbols are different when two strings have the equal length [30]. In the alphabet Σ = {A, C, G, T}, there exists a set S with length n and size of $\left|S\right|=4^{n}$. A subset C⊆S and let *u*, *v* any two codes in the C satisfy [31]:

(1) τ(u,v)≥d

*d* is a positive integer, *τ* is the constraint criteria (or criterion) for designing DNA coding. In this paper, τ is denoted as the Hamming distance.

#### 2.1.1. Sequences-Sequence Hamming Distance (SS)

Sequences-sequence Hamming Distance [31]: for the DNA sequences *u*, *v* with given length *n* (written from the 5′ to the 3′ end), the Hamming distance between *u* and *v* is denoted as *H* (*u*, *v*). The minimal *H* (*u_i_*, *v_j_*) in all DNA sequences is denoted as *SS* (*u_i_*) and it should not be less than parameter *d*,

(2) $$SS(u_{i})=\underset{1\leq j\leq n,j\neq i}{\min}\left\{H\left(u_{i},v_{j}\right)\right\}\geq d$$

#### 2.1.2. Sequences-Complementarity Hamming Distance (SC)

Sequences-complementarity Hamming Distance [31]: for the DNA sequences *u*, *v* with given length *n* (written from the 5′ to the 3′ end), *H* (*u*, *v^C^*) denotes the Hamming distance between *u* and *v^C^*, where *v^C^* is the complementary sequence of *v*. For example, *v* = ACTG, then *v^C^* = CAGT. The minimal *H* (*u_i_*, $v_{j}^{C}$) in all DNA sequences is denoted as *SS* (*u_i_*) and it should not be less than parameter *d*,

(3) $$SC(u_{i})=\underset{1\leq j\leq n,j\neq i}{\min}\left\{H\left(u_{i},v_{j}^{C}\right)\right\}\geq d$$

#### 2.1.3. GC Content

In order to approximate the thermodynamic properties of DNA sequences, *GC* content constraint is used to combine with distance constraint, such as Hamming distance. The percentage of *G* or *C* bases within each DNA is denoted as *GC* content. In this paper, *GC* content is equal to 50%. The *GC* content is denoted as follows:

(4) GC_content=Num_gcn×100%

#### 2.1.4. DNA Coding Rule

Adenine (A), Cytosine (C), Guanine (G) and Thymine (T) are the four elements that make up the whole DNA sequence. When paring, the principle of complementary base pairing is observed, namely A with T and C with G [32]. There is a complementary relationship between 0 and 1 in the binary bit. Similarly, there is a complementary relationship between 01 and 10 as well as 00 and 11. In the previous works, the authors converted binary message to DNA sequences based on the DNA coding rule in Table 1 [6,9,13,14,15,17,23,33,34]. There are eight DNA coding methods to convert binary message to DNA sequences [9,24]. For example, the pixel 65 is firstly transformed into binary bit 01000001 and then 01000001 transformed into DNA sequence ACCA for the first rule.

From the Table 1, the information only is simple transformed between binary and DNA sequence. It does not consider the characters of DNA sequences, especially specific hybridization. So, in this paper, we designed DNA coding that satisfied three constraints above to encrypt image and use this DNA coding to correct the errors.

### 2.2. New DNA Coding Rule for Correcting Errors

As shown above, the DNA sequences used to encode pixels and chaotic orbits should satisfy these constraints to decrease the similarity between DNA sequences and correct the errors. In our previous work, we proposed a dynamic genetic algorithm to design DNA sequence sets which satisfy the combinational constraints [35]. DNA sequence set denotes that any pair of DNA sequences in this set satisfies the combinational constraints. In this paper, we use the DNA coding $A_{4}^{SS+SC+GC}(8,3)=336$, namely the length is equal to 8 and Hamming distance is equal to 3. We randomly select 256 elements from this set to encode the pixels between 0 and 255. In this paper, we denote these 256 DNA sequences as DNA coding rule. Table 2 lists the first 50 DNA coding rule to encrypt image. The whole $A_{4}^{SS+SC+GC}(8,3)$ and DNA coding rule are shown in the Supplement.

### 2.3. Process of Encrypting and Decrypting Image Based on DNA Coding

#### 2.3.1. Encrypting Image

Recently, there are some works on cryptanalysis of encrypting schemes based on chaotic map and DNA coding [36,37,38]. In this paper, in order to improving the security of our encrypting scheme, two logistic maps with different parameters and initial values are chosen to generate pseudorandom sequence. The different parameters and initial values for the Equation (5) are denoted as $\mu_{1}$, $\mu_{2}$, $x_{1}^{1}(0)$ and $x_{1}^{2}(0)$, respectively, where $\mu_{1},\mu_{2}\in [3.9,4]$ and $x_{1}^{1}(0),x_{1}^{2}(0)\in (0,1)$.

(5) $$x_{i+1}=\mu x_{i}(1-x_{i})$$

The detailed of encrypting image is described as follows:

*Step* 1. The key with 16 elements is randomly generated as the initial key and the initial key is implemented XOR operation with every pixel value of the plain image. The result of XOR operation is regard as the relating key;
*Step* 2. According to initial condition of logistic maps, namely two parameters $\mu_{1}$, $\mu_{2}$ and two initial value $x_{1}^{1}(0)$, $x_{1}^{2}(0)$, the relating key is evenly dividing relating key into four parts. These logistic maps are to iterate for 100 times to get rid of the transient effect of chaotic systems;
*Step* 3. The logistic maps are continuingly iterated base on the number of pixels, namely one map for the half number and the pseudorandom sequence consists of the logistic chaotic orbits;
*Step* 4. In order to permute the plain image, the chaotic orbits are sorted in ascending order. This operation (permutation) only changes the location of pixels of plain image;
*Step* 5. The XOR operation is implemented between the pixels of the permuted image and the pseudorandom sequence from the logistic maps. This operation (diffusion) only changes the value of pixels of digital image;
*Step* 6. According to the new DNA coding rule, the encrypted image is encoded by DNA coding;
*Step* 7. Outputting the encrypted image.

**Figure 1:**
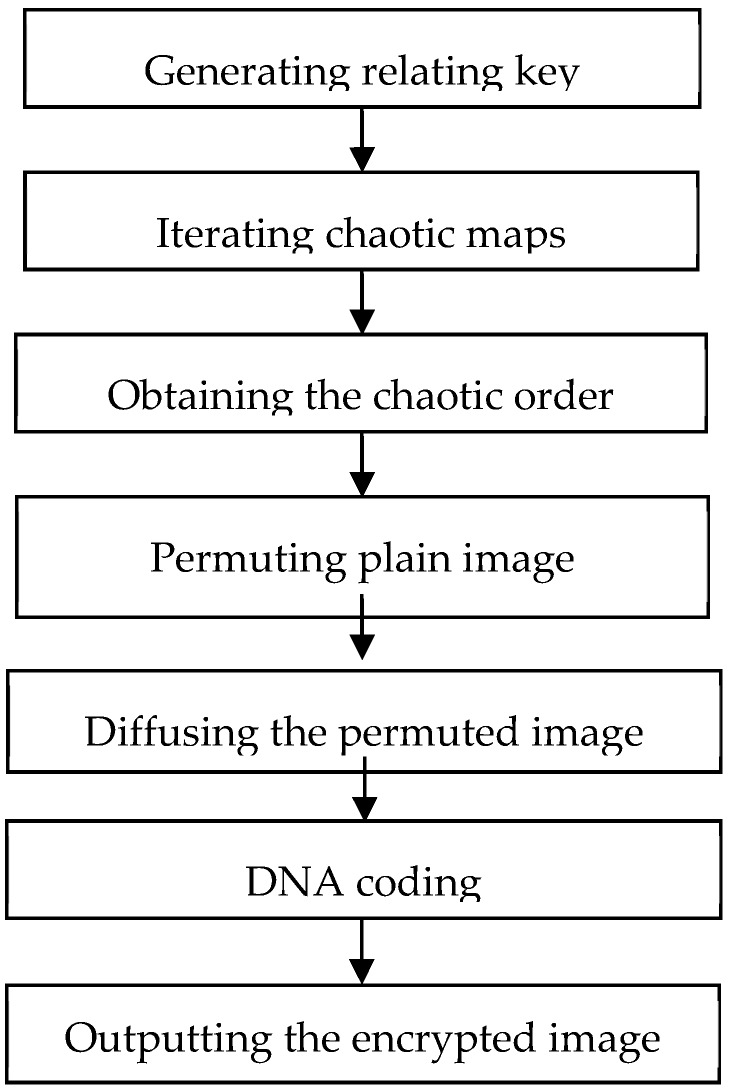
The flowchart of encrypting image.

The flowchart of encrypting image is illustrated in Figure 1.

#### 2.3.2. Decrypting Image

The decryption process is similar to that of encryption procedure in the reversed order. It can be briefly stated as follows:

*Step* 1. According to the same relating key, the chaotic maps are to iterate for 100 times to get rid of the transient effect;
*Step* 2. The chaotic orbits are regenerated based on the same parameters and initial values as well as the encryption process;
*Step* 3. Decoding the cipher image based on the DNA coding rule;
*Step* 4. The XOR operation is implemented between the pixels of the cipher image and the pseudorandom sequence from the logistic maps and the permuted image is recovered;
*Step* 5. According to the order of chaotic sequences, the plain image is recovered from the permuted image;
*Step* 6. Outputting the plain image.

Note that the permutation–diffusion architecture is widely used into image encryption based on chaotic map and DNA coding. So, the whole architecture of the proposed method is the permutation–diffusion. However, the previous works are mainly to convert the pixel value into an 8-bit binary sequence and then perform a simple one-to-one correspondence between the binary (or ASCII codes) and the DNA sequence without the function of error correction. For example, the binary sequence of the pixel value 1 is 00000001 and the corresponding DNA sequence is AAAC (A for 00, C for 01, G for 10 and T for 11). In this paper, a DNA coding scheme with the function of error correction is proposed, where the pixel value of image is directly corresponded to a piece of DNA sequence with the function of error correction.

## 3. Experiment and Simulation

In order to resist the brute-force attack, the key space must be large enough for a secure image cryptosystem. 16 elements make up the key in our paper, $key=\{x_{i}\},i=1,2,\ldots ,16,x_{i}\in [0,255]$. It is sufficiently large to ensure the security of digital image when the key space reaches to $2^{128}\approx 3.4\times 10^{38}$. All the following experiment have the same size for key space.

### 3.1. Key Sensitivity

The test of key sensitivity can be stated as follows:

*Step* 1. Generating the key 123456789012345 and using this key to encrypt the test images;
*Step* 2. Generating another key—123456789012346—with a slight difference and using this key to encrypt the same test image;
*Step* 3. Calculating the difference between different cipher images.

**Figure 2:**
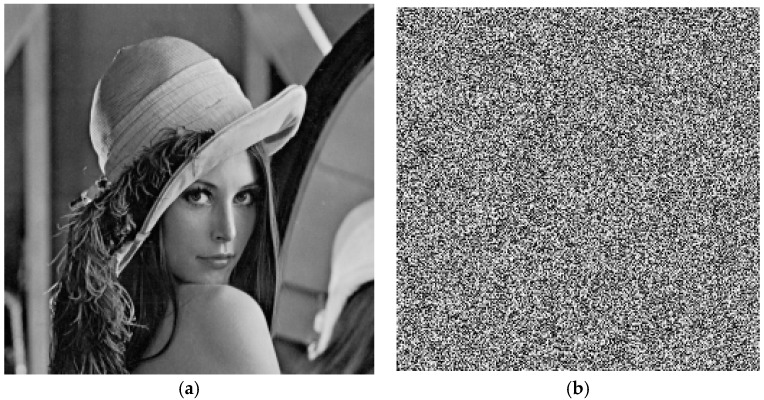

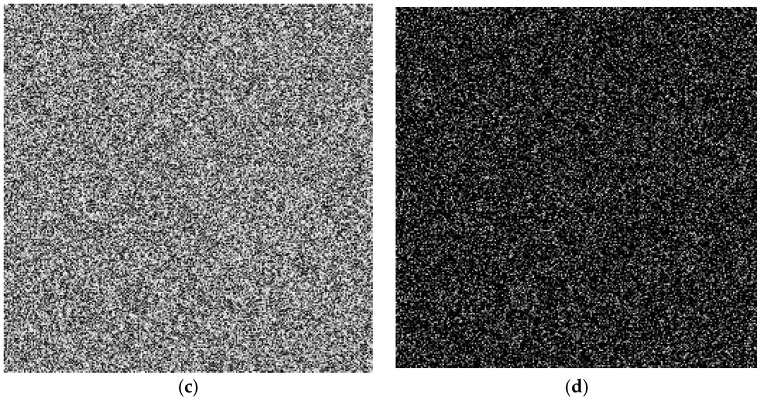
Key sensitivity for Lena. (**a**) Plain-image of Lena; (**b**) Encrypted image by key: 123456789012345; (**c**) Encrypted image by key: 123456789012346; (**d**) Difference image.

**Figure 3:**
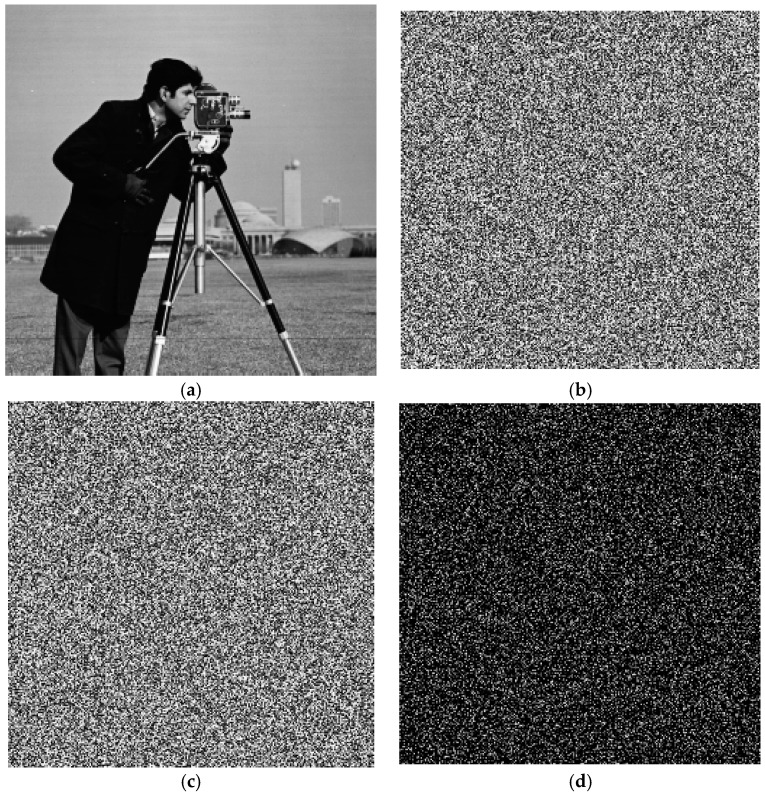
Key sensitivity for Cameraman. (**a**) Plain-image of Cameraman; (**b**) Encrypted image by key: 123456789012345; (**c**) Encrypted image by key: 123456789012346; (**d**) Difference image.

**Figure 4:**
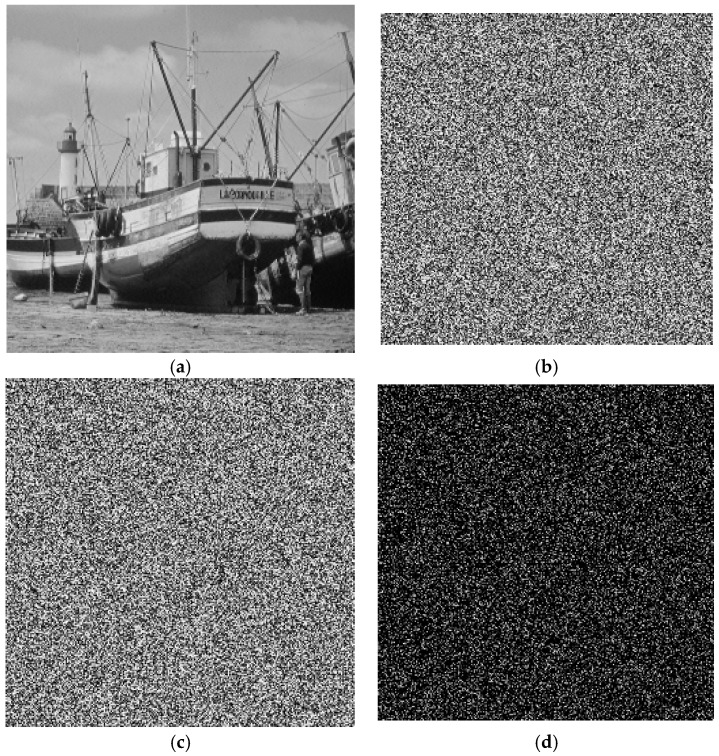
Key sensitivity for Boat. (**a**) Plain-image of Boat; (**b**) Encrypted image by key: 123456789012345; (**c**) Encrypted image by key: 123456789012346; (**d**) Difference image.

From the results, although the two different keys are only slightly different—by one bit—the cipher image with the key 123456789012345 is 99.63% different from the cipher image with the key 123456789012346. Figure 2 shows the results of test image Lena. For the same keys of Cameraman, there is 99.59% difference shown in Figure 3. There is 99.55% difference for Boat shown in Figure 4.

### 3.2. Statistical Analysis

The statistical characteristics of digital image can be exploited to attack the encryption system. The correlation of two adjacent pixels, as one of statistical characteristics of digital image, is the main aspect of statistical attack. 1000 pairs of adjacent pixels are respectively selected from vertical pixels, horizontal pixels and diagonal pixels. The correlation coefficient of each pair is calculated by the following formulas [1]:

(6) cov(x,y)=E{(x−E(x))(y−E(y))}

(7) $$r_{xy}=\frac{\mathrm{cov}(x,y)}{\sqrt{D(x)}\sqrt{D(y)}}$$

where x and y are grey-scale values of two adjacent pixels in the image. As digital image consists of discrete pixels, we adapt the following discrete formulas for calculating the correlation:

(8) $$E(x)=\frac{1}{N}\sum_{i=1}^{N}x_{i}$$

(9) $$D(x)=\frac{1}{N}\sum_{i=1}^{N}{\left(x_{i}-E(x)\right)}^{2}$$

(10) $$\mathrm{cov}(x,y)=\frac{1}{N}\sum_{i=1}^{N}\left\{(x_{i}-E(x))(y_{i}-E(y)\right)\}$$

Table 3 shows the results of horizontal, vertical and diagonal directions. The values outside the brackets indicate the correlation between two adjacent pixels for three different plaintext images and the correlation between the cipher text images is indicated in the brackets. From the experimental results, the proposed algorithm greatly reduces the correlation between pixel values of horizontally, vertically and diagonally adjacent images and improves the ability to resist statistical attacks.

### 3.3. Differential Attack

Number of pixels change rate (NPCR) and Unified average changing intensity (UACI) are the common quantitative criteria for image cryptosystem to evaluate the property of resisting differential attack.

The NPCR and UACI are defined as follows [39,40,41]:

(11) $$NPCR=\frac{\sum_{i,j}D(i,j)}{W\times H}\times 100\%$$

(12) $$UACI=\frac{1}{W\times H}\left[\sum_{i,j}\frac{\left|C_{1}(i,j-C_{2}(i,j))\right|}{255}\right]\times 100\%$$

where $C_{1}$ and $C_{2}$ denotes two different cipher images. These cipher images only have one pixel difference. $C_{1}(i,j)$ and $C_{2}(i,j)$ respectively denote the pixel values at the same point (i,j) of $C_{1}$ and $C_{2}$; *H* and *W* are respectively the height and width of the image; $C_{1}(i,j)$ and $C_{2}(i,j)$ determine the value of D(i,j), namely, if $C_{1}(i,j)=C_{2}(i,j)$ then D(i,j)=0 otherwise, D(i,j)=1.

The comparing results of NPCR and UACI list in the Table 4, where the image cryptosystem adapts the permutation—diffusion architecture with only one round.

The average of ten trials for our method is listed the table. According to the comparison, it shows that our method has higher security.

## 4. Correcting Errors

**Figure 5:**
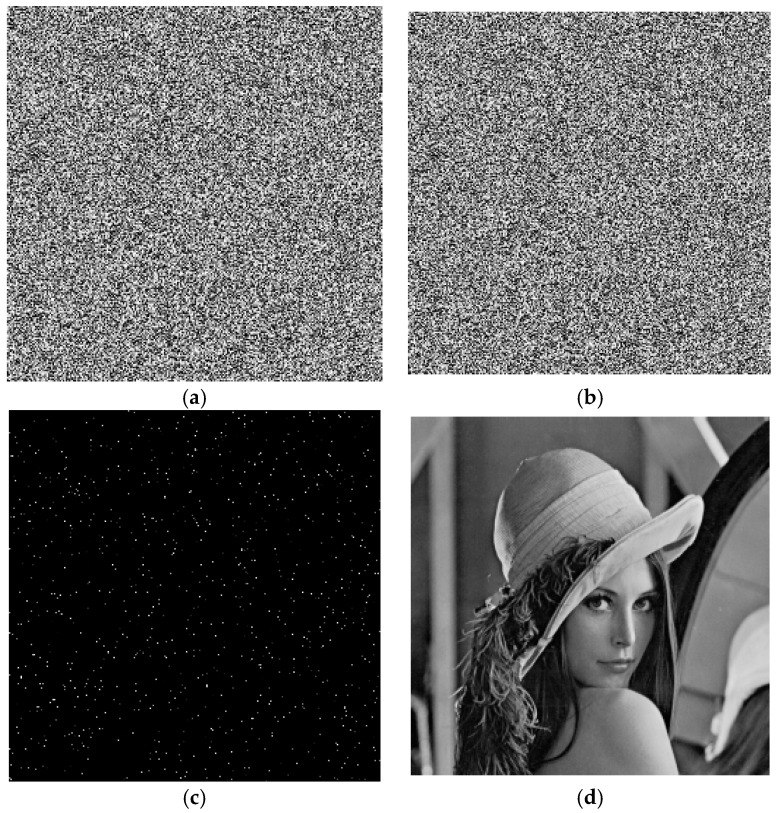
Correcting errors. (**a**) Containing errors image; (**b**) image after correcting errors; (**c**) Difference image; (**d**) Decrypted image after correcting errors.

In this chapter, we simulated the process of correcting errors. First, we encode the cipher image to DNA sequences and randomly change 1000 bases. Each DNA sequence encoded pixel only change one base. Figure 5 shows the effect of correcting errors. Figure 5a shows the encrypted image contain 1000 errors. Figure 5b shows the image after correcting errors by Hamming code. Figure 5c shows the difference between Figure 5a and Figure 5b. Figure 5d shows the decrypted image after correcting errors. The experimental results express that the proposed method could effectively correct the errors and improve the accuracy of hybridization reaction.

Note that if the changed DNA sequence does not match the according to the DNA coding rule, we compulsively set this DNA sequence correspond to the pixel 255. For example, the pixel 16 match DNA sequence GCCTATCT according to DNA coding rule. If the third base is changed, namely GCGTATCT, there will be no pixel match this changed DNA sequence. So, we set GCGTATCT to correspond to pixel 255.

## 5. Conclusions

In this paper, in order to improve the accuracy of DNA computing, we propose a novel method which could decrease the similarity of DNA sequences in DNA computing as well as correct errors from the process of image encryption and decryption. We first analyze the characteristic of DNA hybridization reaction and introduce the combinatorial constraints, namely Sequences-sequence Hamming Distance, Sequences-complementarity Hamming Distance and GC content, to design DNA coding. Then we use the chaotic map to generate pseudo-random sequences and encrypt the plain image by the permuting-diffusing architecture. Finally, we propose a novel DNA coding rule to encode the encrypted image. The experimental results show our method could be used to correct errors in image encryption based on DNA coding.

Bio-inspired computing models, such as membrane computing models [44,45,46,47,48,49,50], may provide intelligent methods for Image Encryption. As well, DNA coding strategies can provide biological ways in solving chemical information processing problems.

## Figures and Tables

**Table 1:** DNA coding list [9,24].

	1	2	3	4	5	6	7	8
A	01	01	00	00	10	10	11	11
T	10	10	11	11	01	01	00	00
C	00	11	01	10	00	11	01	10
G	11	00	10	01	11	00	10	01

**Table 2:** The first 50 DNA coding rule.

Pixel	DNA Coding	Pixel	DNA Coding	Pixel	DNA Coding	Pixel	DNA Coding	Pixel	DNA Coding
0	ATCATGCC	1	CTCGATCA	2	GCTCTTCT	3	AGTGGGAT	4	ACTCTCTG
5	AATCTGCG	6	ACTCACGT	7	CTTCCAAC	8	GCTTCTAG	9	TAGGAGGT
10	GATCGACT	11	TAACGCTG	12	TAAGCGGA	13	CTGTGATC	14	CCCTAATC
15	TGGAAGGA	16	TACTACCG	17	CTTATGGG	18	TCAGCAAG	19	CGACTTCT
20	AGTGTCGA	21	TGCGATTC	22	CAACGACA	23	GATCTGTC	24	GCCAACTA
25	ATGAGGGA	26	TAGAACGG	27	CCGTAACA	28	TAGACTGC	29	GCTGGATT
30	GTGAGTCA	31	TCATGGAC	32	ACCACTAC	33	TCCTAAGG	34	GGCTAAAG
35	CCAACTGA	36	TCGTCTTG	37	TTGGGAAC	38	AATAGCCC	39	CTGTCGAA
40	CCCCATAT	41	AACCTCTC	42	GGTTTACG	43	GCAGAAGA	44	TAGAGGAG
45	GAAAGGGA	46	ATCGACGA	47	GCAAGTAC	48	TCAGACAC	49	CTTGGTTG

**Table 3:** The correlation coefficient of adjacent pixels.

	Horizontal	Vertical	Diagonal
Lena	0.9727(0.0073)	0.9481(0.0058)	0.9250(−0.0091)
Cameraman	0.9561(−0.0053)	0.9213(−0.0062)	0.9145(−0.0059)
Boat	0.9334(0.0006)	0.9249(0.0009)	0.8891(−0.0002)

**Table 4:** The value of NPCR and UACI for Lena.

	NPCR	UACI
Proposed algorithm	99.57%	32.38%
Wang’s work [42]	44.27%	14.874%
Gupta’s work [43]	99.62%	17.30%

## Supplementary Materials

The supplementary materials are available online.
