# Supplementary material for: Correcting Errors in Image Encryption Based on DNA Coding
Source: Molecules. 2018 Jul 27;23(8):1878. doi: 10.3390/molecules23081878 (PMC6222733; doi:10.3390/molecules23081878)
Supplement: Supplementary file 1 [file molecules-23-01878-s001.pdf]

Supplement

This supplement lists all DNA coding used into this paper.

$A_4^{SS+SC+GC}(8,3)=336$

1	ATCATGCC
2	CTCGATCA
3	GCTCTTCT
4	AGTGGGAT
5	ACTCTCTG
6	AATCTGCG
7	ACTCACGT
8	CTTCCAAC
9	GCTTCTAG
10	TAGGAGGT
11	GATCGACT
12	TAACGCTG
13	TAAGCGGA
14	CTGTGATC
15	CCCTAATC
16	TGGAAGGA
17	TACTACCG
18	CTTATGGG
19	TCAGCAAG
20	CGACTTCT
21	AGTGTCGA
22	TGCGATTC
23	CAACGACA
24	GATCTGTC
25	GCCAACTA
26	ATGAGGGA
27	TAGAACGG
28	CCGTAACA
29	TAGACTGC
30	GCTGGATT
31	GTGAGTCA
32	TCATGGAC
33	ACCACTAC
34	TCCTAAGG
35	GGCTAAAG
36	CCAACTGA
37	TCGTCTTG
38	TTGGGAAC
39	AATAGCCC

40	CTGTCGAA
41	CCCCATAT
42	AACCTCTC
43	GGTTTACG
44	GCAGAAGA
45	TAGAGGAG
46	GAAAGGGA
47	ATCGACGA
48	GCAAGTAC
49	TCAGACAC
50	CTTGGTTG
51	TAGCATCC
52	GATACTCG
53	AATGCCCT
54	AACCCTAG
55	CACACGTA
56	GCCTATCT
57	GAGCTCTA
58	TGAAGCGA
59	TAACCGCT
60	GAACTCGT
61	GAGGGAAA
62	TGTCTAGC
63	CGAACCTT
64	AGGAATCC
65	AGGTGGAA
66	TACCCAGT
67	TCACGTAG
68	TATCCGTG
69	TTCGAACC
70	AGCTCTGA
71	GTTGCACA
72	AAGCCGTT
73	TCACATGC
74	CCCAAGAA
75	CTCAGTCT
76	ATCGGTAG
77	ACGGACAA
78	CCAGTTCA
79	TTATCCCG
80	GTACGATG
81	TCTACGGT
82	CAACCTTC
83	GTCATCCA

84	ACTACACG
85	CGGTTTTG
86	ACCAACAG
87	ACGCATAC
88	TGACGTGT
89	ATGACCTC
90	CTTGACTC
91	CATTCCGA
92	TGTGAACG
93	TGGCGATA
94	ACGTTGGA
95	ATTCAGGC
96	GTAGTCAG
97	AAGCGTCT
98	TCTCTGGA
99	GACACCTT
100	ACCCTAAC
101	AGCTTAGG
102	TGACATCG
103	GAACAACC
104	ATGCTCGT
105	CACCTGTT
106	AGTCCAAG
107	CGTCATAG
108	AGCTTGTC
109	GCGATAGA
110	TCCCAGTT
111	GCGCATTT
112	GTTCAGCT
113	AGAGGCAA
114	TAAGGTGC
115	CAGACGAT
116	TCGGATGA
117	GAATGAGG
118	CTTCTCAG
119	CGGTGAAT
120	CGCAAAGA
121	ACGTAGTG
122	CACTGCAA
123	TTGGCGTT
124	CAATCCTG
125	CTCGCTTT
126	TGGTTCAG
127	CGCCTAAT

128	GAAACCAG
129	CCTTGATG
130	TGGATTCG
131	ATGTTGCG
132	GCAAAACG
133	TCAGCCTT
134	ACGGAACT
135	AAACGGTC
136	GCCCTTAA
137	GGACTACA
138	CCATAGAG
139	ATAGGGAC
140	AGTCGTGA
141	CCTGTGTT
142	AGGCTTTC
143	CTACATCC
144	AGCACGTT
145	GGAGAGAT
146	CCTTGCAT
147	TGCCACAA
148	CAAAAGGC
149	GGTCACTA
150	AGAACGGA
151	CTGAAGTC
152	CACCTTGA
153	CATTCGCT
154	ATGTGCAG
155	TAAACCCC
156	ACATGCTG
157	CATTTCCG
158	ACCTTGAG
159	GATGGTCA
160	TCTACCTG
161	CTAACTCG
162	GTACCCTT
163	GCATCAAC
164	AAGCAGGA
165	CAGTACAC
166	ATGGAGAG
167	CGTACTTG
168	GGTTTGGT
169	CAAAGCAC
170	CTAAGCTG
171	CAGTTGCA

172	ACTGATCG
173	TTAGGTCG
174	ACTTGCGA
175	GTGGTTCT
176	CCGTCATT
177	TTGAGTGG
178	CATCGCTT
179	CTGGTCAA
180	ATGAGACC
181	CCTCCATA
182	AGATGGCT
183	ACGACGTA
184	TTACACGG
185	TCTGACCA
186	CAAGGGAA
187	CAGACATC
188	CGAATGTG
189	ACGCCAAA
190	TCTGCGAA
191	ATGTCCCA
192	TATCGGGT
193	ACTGCATC
194	CTAGACCT
195	ACACTAGG
196	TCTCGAAC
197	TTCCGACA
198	GTACACCA
199	GTTTCGTG
200	GGGATTGT
201	AGATACCG
202	GTACAGAC
203	GTTAACGG
204	TGATCTCC
205	AAGACCGT
206	TTTTGCCC
207	CAAAGTGG
208	GGTGTTAC
209	GTCGAGTA
210	GTTAGCTC
211	CGATACTC
212	GCTATCAG
213	TTCGTGCA
214	GCAACTCT
215	GGACCAAT

216	AGTTTCCC
217	ACCGATGT
218	AGGAGCTA
219	GAGTGTGT
220	ACACTGCT
221	CTCTGAGT
222	GCAGATAG
223	CGAAGTTC
224	TGAAAGCC
225	GCAATGCA
226	ATCGCAAC
227	ATGCTGAC
228	GACTCTTC
229	AGGACACA
230	CTCACATG
231	GGTAGAAC
232	GGATTGAC
233	CGTAGGTA
234	AAGGAAGC
235	CAGACTCA
236	GTCTAAGC
237	TGGGTACA
238	CACTTAGC
239	GATCCTGA
240	CTTATCCC
241	TAACTGGG
242	AGTAAGGG
243	AGAGTGCA
244	AAAGGGGT
245	CATTGGTC
246	AACTGTCG
247	ATAGCCGT
248	TATGCTCC
249	GATACGAC
250	GTACGTCT
251	ACTTCGCA
252	CGCTATAC
253	TCTGAGTC
254	ACGAATGG
255	GCGAAGAT
256	CGCTTCTT
257	ACGGTAAG
258	ATTGCCAG
259	TCTAGCCT

260	AACTAGCC
261	TCGGGTTT
262	ACTTCCAC
263	TAGCCCAA
264	TACGCATC
265	TACGATGG
266	TATGGGAC
267	GGCGTAAA
268	CAAGGTCT
269	TTCTGGTC
270	CTGCTACT
271	TGGTTGCT
272	ATCTGCCT
273	CCAGGAAT
274	GGTCCTTT
275	AGCAAGAC
276	GAGGCTTT
277	CCTACCAA
278	ACGTTTCC
279	ACAGTGTG
280	ATGGCTTG
281	TTTGTGGC
282	CACATGAC
283	TAAGTGCC
284	CTTTGGAG
285	CGCTTGAA
286	ATCACGAG
287	TGCCTTAG
288	AAGTTCGG
289	TCGTACCT
290	ATAGCACG
291	GACAGTAG
292	GGAATCTC
293	AGTCAGCA
294	ATGTCAGC
295	GTGTGGAT
296	TCGCACTA
297	ACTTGACC
298	GTTTGTGG
299	GAATGTCC
300	TATCACGC
301	CCTGAAAG
302	CATCCGAA
303	GTGCAATC

304 TGTTCGTC

305 GGTGATTG

306 GCAGTACT

307 CGTCTATG

308 TCGGTTAC

309 AACCATGC

310 GCTACTTC

311 TGAGGGTA

312 TACAGGCA

313 GCTGTTGA

314 GTGTCACT

315 GTCCTAGT

316 CACCAACT

317 CGCCATTA

318 ATACCGCA

319 CGTGGAAA

320 GCACTATC

321 CTCCTTAC

322 AGTGCTCA

323 TACACCGA

324 TCGTGAAG

325 TGCTCATG

326 GCAAGAGT

327 GTGTTGTC

328 GCACGAAA

329 ATCCTGGA

330 ATGGGTGT

331 AGCGTCAT

332 CTAGCCTA

333 ATCCTTCG

334 ATCCGTTC

335 AGAATCGG

336 TCTGTACC

0	ATCATGCC	1	CTCGATCA	2	GCTCTTCT	3	AGTGGGAT	4	ACTCTCTG
5	AATCTGCG	6	ACTCACGT	7	CTTCCAAC	8	GCTTCTAG	9	TAGGAGGT
10	GATCGACT	11	TAACGCTG	12	TAAGCGGA	13	CTGTGATC	14	CCCTAATC
15	TGGAAGGA	16	TACTACCG	17	CTTATGGG	18	TCAGCAAG	19	CGACTTCT
20	AGTGTCGA	21	TGCGATTC	22	CAACGACA	23	GATCTGTC	24	GCCAACTA
25	ATGAGGGA	26	TAGAACGG	27	CCGTAACA	28	TAGACTGC	29	GCTGGATT
30	GTGAGTCA	31	TCATGGAC	32	ACCACTAC	33	TCCTAAGG	34	GGCTAAAG
35	CCAACTGA	36	TCGTCTTG	37	TTGGGAAC	38	AATAGCCC	39	CTGTCGAA
40	CCCCATAT	41	AACCTCTC	42	GGTTTACG	43	GCAGAAGA	44	TAGAGGAG
45	GAAAGGGA	46	ATCGACGA	47	GCAAGTAC	48	TCAGACAC	49	CTTGGTTG

50	TAGCATCC	51	GATACTCG	52	AATGCCCT	53	AACCCTAG	54	CACACGTA
55	GCCTATCT	56	GAGCTCTA	57	TGAAGCGA	58	TAACCGCT	59	GAACTCGT
60	GAGGGAAA	61	TGTCTAGC	62	CGAACCTT	63	AGGAATCC	64	AGGTGGAA
65	TACCCAGT	66	TCACGTAG	67	TATCCGTG	68	TTCGAACC	69	AGCTCTGA
70	GTTGCACA	71	AAGCCGTT	72	TCACATGC	73	CCCAAGAA	74	CTCAGTCT
75	ATCGGTAG	76	ACGGACAA	77	CCAGTTCA	78	TTATCCCG	79	GTACGATG
80	TCTACGGT	81	CAACCTTC	82	GTCATCCA	83	ACTACACG	84	CGGTTTTG
85	ACCAACAG	86	ACGCATAC	87	TGACGTGT	88	ATGACCTC	89	CTTGACTC
90	CATTCCGA	91	TGTGAACG	92	TGGCGATA	93	ACGTTGGA	94	ATTCAGGC
95	GTAGTCAG	96	AAGCGTCT	97	TCTCTGGA	98	GACACCTT	99	ACCCTAAC
100	AGCTTAGG	101	TGACATCG	102	GAACAACC	103	ATGCTCGT	104	CACCTGTT
105	AGTCCAAG	106	CGTCATAG	107	AGCTTGTC	108	GCGATAGA	109	TCCCAGTT
110	GCGCATTT	111	GTTCAGCT	112	AGAGGCAA	113	TAAGGTGC	114	CAGACGAT
115	TCGGATGA	116	GAATGAGG	117	CTTCTCAG	118	CGGTGAAT	119	CGCAAAGA
120	ACGTAGTG	121	CACTGCAA	122	TTGGCGTT	123	CAATCCTG	124	CTCGCTTT
125	TGGTTCAG	126	CGCCTAAT	127	GAAACCAG	128	CCTTGATG	129	TGGATTCG
130	ATGTTGCG	131	GCAAAACG	132	TCAGCCTT	133	ACGGAACT	134	AAACGGTC
135	GCCCTTAA	136	GGACTACA	137	CCATAGAG	138	ATAGGGAC	139	AGTCGTGA
140	CCTGTGTT	141	AGGCTTTC	142	CTACATCC	143	AGCACGTT	144	GGAGAGAT
145	CCTTGCAT	146	TGCCACAA	147	CAAAAGGC	148	GGTCACTA	149	AGAACGGA
150	CTGAAGTC	151	CACCTTGA	152	CATTCGCT	153	ATGTGCAG	154	TAAACCCC
155	ACATGCTG	156	CATTTCCG	157	ACCTTGAG	158	GATGGTCA	159	TCTACCTG
160	CTAACTCG	161	GTACCCTT	162	GCATCAAC	163	AAGCAGGA	164	CAGTACAC
165	ATGGAGAG	166	CGTACTTG	167	GGTTTGGT	168	CAAAGCAC	169	CTAAGCTG
170	CAGTTGCA	171	ACTGATCG	172	TTAGGTCG	173	ACTTGCGA	174	GTGGTTCT
175	CCGTCATT	176	TTGAGTGG	177	CATCGCTT	178	CTGGTCAA	179	ATGAGACC
180	CCTCCATA	181	AGATGGCT	182	ACGACGTA	183	TTACACGG	184	TCTGACCA
185	CAAGGGAA	186	CAGACATC	187	CGAATGTG	188	ACGCCAAA	189	TCTGCGAA
190	ATGTCCCA	191	TATCGGGT	192	ACTGCATC	193	CTAGACCT	194	ACACTAGG
195	TCTCGAAC	196	TTCCGACA	197	GTACACCA	198	GTTTCGTG	199	GGGATTGT
200	AGATACCG	201	GTACAGAC	202	GTTAACGG	203	TGATCTCC	204	AAGACCGT
205	TTTTGCCC	206	CAAAGTGG	207	GGTGTTAC	208	GTCGAGTA	209	GTTAGCTC
210	CGATACTC	211	GCTATCAG	212	TTCGTGCA	213	GCAACTCT	214	GGACCAAT
215	AGTTTCCC	216	ACCGATGT	217	AGGAGCTA	218	GAGTGTGT	219	ACACTGCT
220	CTCTGAGT	221	GCAGATAG	222	CGAAGTTC	223	TGAAAGCC	224	GCAATGCA
225	ATCGCAAC	226	ATGCTGAC	227	GACTCTTC	228	AGGACACA	229	CTCACATG
230	GGTAGAAC	231	GGATTGAC	232	CGTAGGTA	233	AAGGAAGC	234	CGCTATAC
235	GTCTAAGC	236	TGGGTACA	237	CACTTAGC	238	GATCCTGA	239	CTTATCCC
240	TAACTGGG	241	AGTAAGGG	242	AGAGTGCA	243	AAAGGGGT	244	CATTGGTC
245	AACTGTCG	246	ATAGCCGT	247	TATGCTCC	248	GATACGAC	249	GTACGTCT
250	ACTTCGCA	251	CGCTATAC	252	TCTGAGTC	253	ACGAATGG	254	GCGAAGAT
255	CGCTTCTT								
